# Jail Diversion for Persons with Serious Mental Illness Coordinated by a Prosecutor's Office

**DOI:** 10.1155/2017/7917616

**Published:** 2017-12-03

**Authors:** Kenneth J. Gill, Ann A. Murphy

**Affiliations:** Rutgers University School of Health Professions, Newark, NJ, USA

## Abstract

Persons with serious mental illnesses (SMI) are involved in the criminal justice system at a disproportionately higher rate than the general population. While the exact causes remain unclear, it is accepted that a comprehensive strategy including mental health treatment is needed to reduce recidivism. This paper describes a unique jail diversion program coordinated by a county prosecutor's office in which individuals were diverted towards mental health services including case management, community-based services, and housing supports. Outcomes were studied over a five-year period, beyond the typical 12- to 24-month follow-up in other studies. Individuals who completed the program, compared to those who did not complete it, were at lower risk for being rearrested, arrested fewer times, and incarcerated fewer days. Gains were moderated by previous criminal justice involvement and substance use but, nevertheless, were maintained despite severity of history. The strongest gains were seen while the individual was still actively enrolled in the diversion services and these outcomes were maintained for up to four years. These findings suggest that completion of a jail diversion program facilitated by a prosecutor's office can lower recidivism and days incarcerated. Further research is needed to assess the unique contribution of prosecutor office facilitation.

## 1. Introduction

The high rate of serious mental illnesses among people who are currently incarcerated poses moral, logistic, and financial concerns. A synthesis of the available data concludes that accurate prevalence rates of mental illnesses in jails and prisons are difficult to determine [1]. Challenges regarding operational definitions of mental illness, as well as the type of incarceration facility, lead to inconsistency in estimations [2]. While these challenges make it difficult to determine a specific prevalence rate, a recent systematic review concludes that for numerous mental illnesses the prevalence rate is elevated among incarcerated populations compared to the general population, and sometimes this rate is substantially higher [2].

People with mental illnesses in the jail and prison systems often go without proper mental health treatment [1]. Additionally, they serve longer sentences and their incarcerations are more costly, perhaps due to the lack of adequate treatment [3]. They are also at higher risk for repeat incarcerations and return to prison more quickly than those without a mental illness [4, 5].

The causes of criminal justice involvement among people with mental illnesses are not entirely clear. One hypothesis is that the behaviors that lead to the arrest of people with mental illnesses are, in large part, a consequence of their mental illness or related circumstances [6]. This is often referred to as the “criminalization” of people with mental illnesses. Illness-related behaviors that may have previously resulted in hospitalization now result in criminal charges due to the larger number of people with serious mental illness living outside of the hospital and the reduced availability of hospitalization when they do become symptomatic [7, 8]. An alternative hypothesis is that the factors predictive of criminal behavior among people with mental illness are the same as those for the general population, but people with mental illnesses have higher levels of these risk factors [9] and, among a minority, a greater propensity for violence and criminal behavior [10]. This “criminogenic” factors hypothesis suggests that nonclinical risk factors contribute to criminal justice involvement for people with mental illnesses [9, 11, 12].

Evaluation of these hypotheses has found support for both perspectives. The criminalization hypothesis is supported by evidence that mental illness symptoms are directly related to criminal behavior for a minority of individuals [9, 11, 13, 14]. Additionally, several clinical factors (i.e., diagnosis, symptoms, and medication nonadherence) have been found to be predictive of arrest [10, 15]. Findings suggest that between 10 and 20% of criminal behavior among people with mental illness is at least somewhat related to illness symptoms [13, 14].

Alternatively, there is evidence supportive of general risk factors that are related to criminal behavior for individuals with and without mental illnesses. Factors such as prior criminal history, criminal thinking patterns, substance abuse, antisocial traits, association with others involved in criminal activities, psychosocial factors, and sociodemographic variables are predictive of criminal behavior regardless of mental illness [9, 10, 12, 15, 16]. It is important to note that substance abuse plays a significant role in criminal behavior among all people but may be of particular importance for people with mental illnesses [11, 13].

Despite a lack of consensus regarding the causes of increased criminal justice involvement, there have been a number of efforts to divert persons with serious mental illnesses away from the criminal justice system and towards the mental health care system [17]. Ringhoff and colleagues [12] have suggested that “access to evidence-based mental health services remains a crucial first step in a comprehensive strategy” (p. 12) to reduce criminal justice involvement. Other modifiable risk factors, such as homelessness, substance abuse, and employment, also need to be targeted in efforts to reduce subsequent arrests [12].

A variety of diversionary criminal justice initiatives have been developed that vary in their structure and the points within the criminal justice system from which they operate [17, 18]. The diversion initiatives can be generally divided into prebooking and postbooking approaches. Prebooking diversion redirects the individual to treatment services at the point of initial contact with law enforcement, prior to the laying of charges [17, 18]. Examples of prebooking diversion programs include Crisis Intervention Teams and Mobile Crisis Units. Postbooking diversion models redirect the individual after arrest and booking of charges. Diversion in postbooking programs can occur in place of prosecution or as a condition of a reduced sentence or charge [17]. Jail-based diversion is one of three general types of postbooking diversion (Broner, Borum, and Gawley, 2002 as cited in [17]). In a jail-based diversion program, individuals in jail who may have a mental illness are identified and evaluated and, if found eligible for diversion, are connected with mental health treatment [17, 18]. This process requires the agreement of the prosecutor, judge, and defense attorney [18].

While these programs have continued to proliferate over time, the research on the criminal justice outcomes of these diversionary programs has been mixed [17, 18]. A structured review of 21 published jail diversion outcomes studies found that there was very little evidence for a reduction in criminal activity or the rate of recidivism, but solid evidence that the amount of jail time served was reduced as a result of diversion [18]. Another review of 11 jail-based diversion programs came to somewhat different conclusions, finding that there was a high degree of effectiveness in reducing recidivism and moderate effectiveness in reducing the number of days incarcerated [17]. What accounts for these differing findings is not entirely clear, but it is likely impacted by the higher level of methodological rigor required for inclusion in the Sirotich [18] review. In order to be included in Sirotich's review the study had to include a comparison group, whereas Lange and colleagues included studies without comparison groups. However, Sirotich [18] also highlighted that failure to account for covariates, high attrition rates, and small sample sizes may also contribute to inconsistencies in the outcomes.

This article seeks to add to the existing literature on jail diversion programs by evaluating the effectiveness of a program administered by a county prosecutor's office over a five-year follow-up period. Previous literature has only been able to report on recidivism and jail days up to 24 months after diversion. This evaluation is able to consider the longer-term impact of a diversion program. Additionally, the program evaluated is unique in that it originated from and is housed within the county prosecutor's office. While other programs rely on the involvement of the prosecutor, few are run out of the prosecutor's office. As a prosecutor-based diversion program, its unique feature was that the prosecutor's office itself coordinated the diversion effort, working with the court, defense counsel, and mental health providers. When these programs are not based in the prosecutor's office, typically defense counsel and a behavioral health care provider have to advocate with a potentially reluctant prosecutor to consider this option. By the nature of the court system, this is necessarily an adversarial process. Having a prosecutor's office philosophically committed to alternatives for persons with serious mental illness will result in a less adversarial approach to diverting the individual from incarceration to treatment alternatives. Additionally, the prosecutor's office has a significant amount of discretion regarding the disposition of the charge and can intervene earlier in the adjudication process. All of these factors help to ensure that appropriate individuals are considered for diversion and that diversion occurs as quickly as possible, potentially limiting the time an individual has to spend in jail.

## 2. Method

### 2.1. Participants

The jail diversion program began in April 2006 with the evaluation period concluding in April 2011. One hundred and thirty-one individuals participated in the program. Complete data sets were available for 125 participants and were included in this analysis. Cases with missing data for a particular analysis were excluded. The average age of participants was 40 years old (SD = 11.98) and the sample was 72.5% males (*n* = 95). Thirty-three percent of individuals (*n* = 44) had a diagnosis of schizophrenia or schizoaffective disorder, 40% (*n* = 52) had bipolar disorder, 17% (*n* = 22) had depression, 5% (*n* = 6) had anxiety, and 5% (*n* = 7) had other diagnoses. Fifty-seven percent (*n* = 74) also had a cooccurring drug and/or alcohol diagnosis.

### 2.2. Procedure

This jail diversion program was unique in that it diverted and supervised individuals at any stage in the criminal justice process, from prearrest through postsentencing. Referrals were made to the prosecutor's office from a variety of sources including jail, law enforcement, defense attorneys, the courts, family members, and mental health professionals. Before enrollment in the program, an assistant prosecutor conducted an evaluation to determine legal appropriateness. For an individual to be legally appropriate the charges had to be of a nonviolent nature and the individual could not have multiple prior convictions for indictable offenses (felonies). Violent offenses were considered on a case-by-case basis. If found legally appropriate and permission was secured from defense counsel, a clinical evaluation was conducted by an associated hospital and then by a community-based mental health program. If the individual was deemed appropriate for community-based services and was willing to participate in the program, a treatment plan was developed and incorporated into the disposition of the case. Mental health treatment could be a condition of bail, permitting the defendant's release without the need to post money, or could be a condition of probation, allowing a defendant to avoid a county jail or state prison sentence.

### 2.3. Measures

The Union County Prosecutor's Office compiled data regarding the types of arrests and convictions, jail/prison days, dates of jail admission and release, and total jail days (both lifetime prior to diversion and that from diversion through April 2011, allowing for up to 60 months of follow-up). County, state, and federal databases of arrests and incarcerations were accessed by the prosecutor's office to collect this data. Various measures of criminal justice outcomes after diversion were collected including arrests for different categories of charges, number of arrests, and community tenure before arrest regardless of arresting jurisdiction.

The community-based mental health programs provided data regarding program participation including admission and discharge data and whether the prescribed program of treatment was completed. All participants received case management services, assertive outreach, and psychotropic medication monitoring if needed. In addition, housing services were provided to those who were homeless or at risk for homelessness. Other outpatient services and inpatient care were provided. Unfortunately, the details regarding the amount and type of services provided to individuals were not available as part of the database made available by the county prosecutor's office for this evaluation.

Global level of functioning (GLOF) was also assessed at baseline and six months into the program. GLOF is a measure developed by the New Jersey Division of Mental Health Services (NJ DMHS) to provide an overall assessment of community functioning and coping with symptomatology. Persons with a score of three or less are typically in institutional settings. Those with scores of four through six are increasing their level of independent functioning and coping, although they may occasionally be hospitalized or in supervised settings. People with a score of seven or more are completely independent. While the GLOF is a required measure by NJ DMHS, there is limited reporting of its psychometric properties. There is no available data on the measure's interrater reliability. There is, however, support for its validity from one study [19]. In a sample of over 10,000 participants, four subscales of the Specific Level of Functioning scale (SLOF; [20]) were found to be positively correlated with the GLOF: personal care skills, work skills, daily activity functioning, and interpersonal relationships. Also, the GLOF was significantly related to type of residence, with higher ratings associated with independent living settings and lower ratings associated with recent hospitalization. The two strongest associations with GLOF ratings were found to be SLOF daily living functioning and SLOF work skills, which together accounted for 44% of the variance [19].

### 2.4. Study Design

Institutional Review Board approval was obtained to analyze this existing data set provided by the Union County, NJ prosecutor's office. This is a naturalistic study in that it was not designed as a research experiment but grew out of a program evaluation of a local innovation. It includes pre-post analyses, between group comparisons, correlational analyses, and survival analysis. The preformed groups of “completers” versus “noncompleters” were used. Completers are those who finished the court-ordered treatment as originally recommended and prescribed by the mental health provider. Their reason for discharge from the program was that they completed the treatment ordered. Noncompleters are those who did not follow through with the prescribed treatment as originally ordered and were discharged for other reasons. Survival analyses tracking changes in the proportions of criminal justice outcomes were conducted for up to five years.

## 3. Results

### 3.1. Nature and Type of Diversions

During the study period, a total of 131 persons were enrolled in the jail diversion program. Each person received both a legal disposition and a court-ordered treatment plan facilitated by a collaborating mental health provider. Treatment included initial evaluation and diagnosis, psychotropic medication monitoring if prescribed, and additional individualized treatments. These diversions took place at a variety of stages. Approximately 27% (*n* = 35) were diverted at the point of prison sentencing, 53.4% (*n* = 70) were diverted from jail stays (i.e., either pretrial or for brief sentences), and 19.8% (*n* = 26) were diverted before arraignment or before trial by the prosecutor's office. The legal disposition of the diversions included reduced sentences (15%), dismissal of charges (13%), downgrading or reduction of charges to a less serious charge (58%), or pretrial interventions (14%).

### 3.2. Pre-Post Enrollment in Jail Diversion

**Figure 1:**
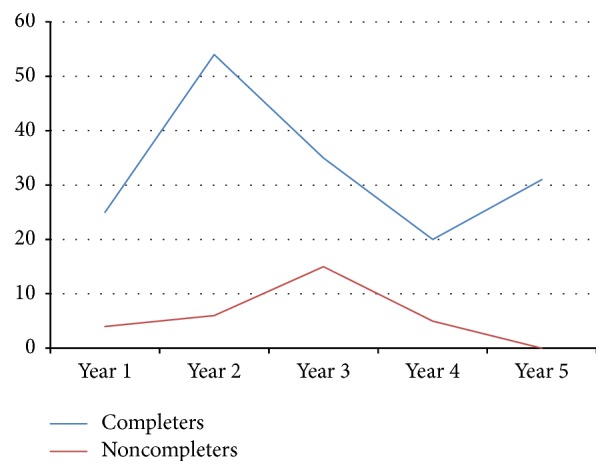
Mean days incarcerated each year of follow-up for completers versus noncompleters.

In the year prior to diversion, participants spent an average of 28.88 (SD = 70) days incarcerated. In the first 12 months after enrollment, this was reduced by 13 days to 15.82 days (*t*(129) = 1.97, *p* = .05). Participants who completed their prescribed program had significantly better outcomes than noncompleters.  Figure 1 presents days incarcerated for the completers compared to noncompleters. In the year prior to enrollment compared to the year after enrollment, those who completed the program dropped from 25 days to 4 days, respectively. Those who did not complete the program reduced their time incarcerated from 33 days to 25 days, which was not significant.

**Figure 2:**
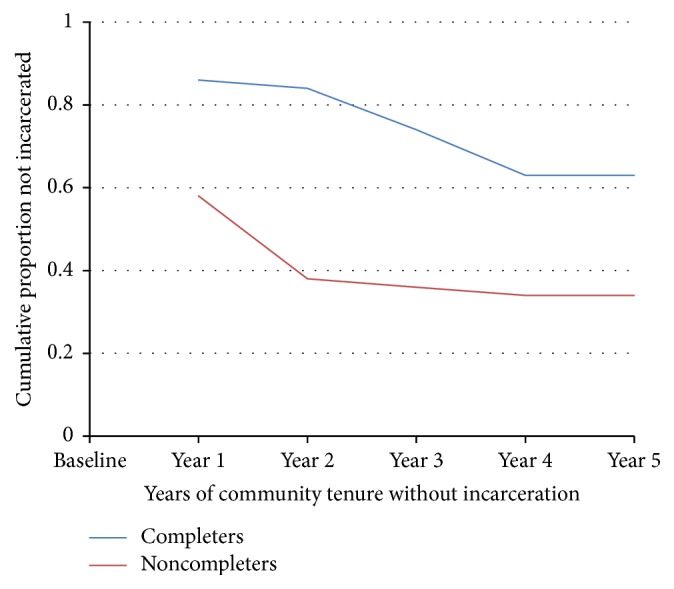
Cumulative proportion of completers versus noncompleters not incarcerated over a 5-year follow-up period.

Compared to the year prior to enrollment, completers reduced their number of days incarcerated in the five subsequent years. Noncompleters only had more days incarcerated in year 2 (13–24 months) than in the year prior to enrollment. However, for each follow-up period, noncompleters spent more days in jail than completers. A survival analysis examined length of community tenure spent unincarcerated as an outcome variable. In the first year, 42% of the noncompleters were reincarcerated compared to 14% among the completers. By the five-year mark, 67% of noncompleters had been incarcerated. In contrast, among those who completed the program, only 37% had been incarcerated even after 5 years. In Figure 2, the differences between completers and noncompleters in the follow-up periods are illustrated, with the upper function (blue line) representing completers and the lower function (red line) representing noncompleters.

**Figure 3:**
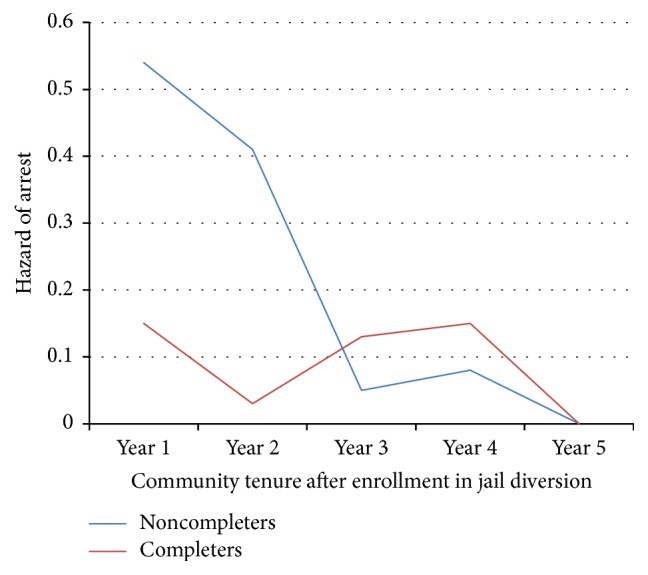
Hazard functions of becoming incarcerated of noncompleters versus completers.

Participants who are noncompleters are at significantly higher risk for reincarceration after entering the jail diversion program through year three (see Figure 3). During that time period, the risk of incarceration is four to five times higher among noncompleters than completers. Around year three, the risk for noncompleters begins to decline and the risk for completers rises modestly. Completers were less likely to be arrested in the follow-up period, and if arrested, it was after significantly longer community tenure. Noncompleters averaged 2.02 annually arrests up to 5 years after enrollment in the jail diversion program, whereas completers averaged 0.63 arrests (*F*(1,124) = 5.62, *p* < .05). The median point at which arrest has occurred for 50% of participants was 19 months for noncompleters, whereas for completers it was 48 months.

**Figure 4:**
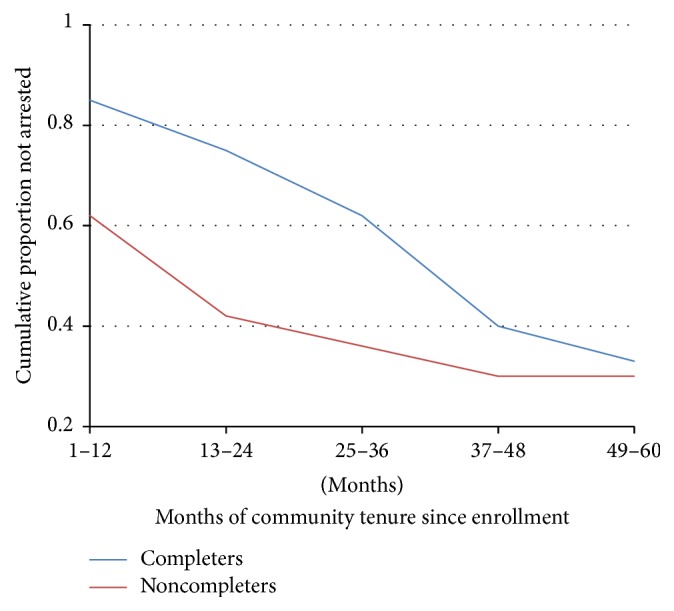
Monthly cumulative proportion of persons (completers versus noncompleters) remaining in community without any arrests over 60 months.

The survival curve illustrates the cumulative proportion of individuals who are not rearrested. In Figure 4, compare the blue line to the red line. Consistently a higher cumulative proportion of program completers are maintained in the community without arrest for four years. In the fifth year, the proportion remaining in the community without any arrest drops to virtually the same for both groups.

**Figure 5:**
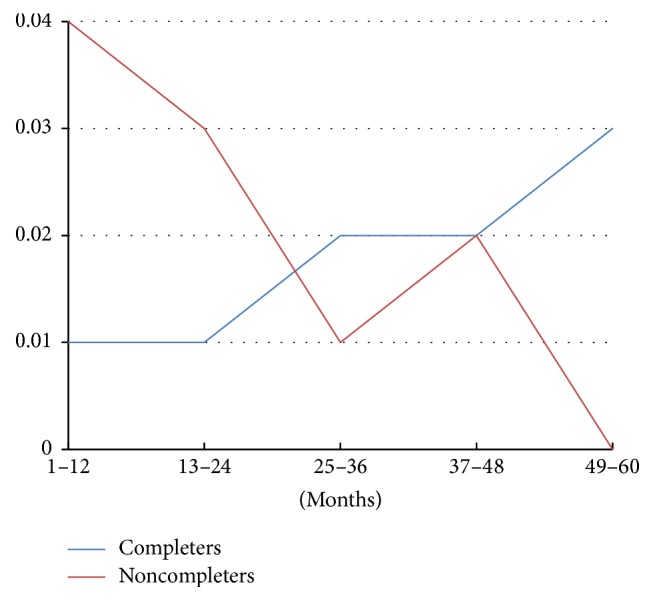
Hazard functions of proportion of arrests of noncompleters versus completers by follow-up month.

In order to better understand the pattern of changes, a hazard function is presented in Figure 5 showing the likelihood of first arrest at each stage of the follow-up period. The darker line represents noncompleters and the lighter line completers. For years 1 through 2, noncompleters are more likely to be arrested; however, in years 3 and 4 it is about comparable. In year 5 completers have a much higher risk of rearrest.

### 3.3. Positive Changes in Global Level of Functioning

GLOF increased significantly to 5.31 from a baseline of 4.76, after 6 months in the jail diversion program (*t*(81) = 6.72, *p* < .001). This indicates significantly increased community integration, better overall functioning, and management of symptoms. There was a larger increase in GLOF among completers (change score = .85, *t*(41) = 8.70, *p* < .001), compared to noncompleters (change score = .25). Nevertheless, this smaller pre-post difference among noncompleters was also statistically significant (*t*(39) = 2.69, *p* = .01).

### 3.4. Characteristics of Completers versus Noncompleters

Noncompleters received, on average, 19 weeks of services less than those who complete the program (38 weeks versus 57 weeks, resp.). Their increase in jail days seems to follow termination from the mental health services, spiking in the second year of follow-up (13–24 months after entry). The reasons for noncompletion of the program varied. Among noncompleters, approximately 51% (*n* = 37) refused services or were noncompliant, another 15% (*n* = 11) were lost to contact, 6% (*n* = 4) were deceased, and the remaining 28% (*n* = 20) were coded by the prosecutor's office as “other” reasons with no further details specified.

### 3.5. Noncompleters and Completers prior to Diversion

In many ways, completers and noncompleters were alike. There were no significant differences in terms of age (*t*(121) = −.314, ns), gender (the majority of each sample was male, *χ*^2^(1) = .092, ns), and the frequency of psychiatric diagnoses (*χ*^2^(2) = 1.74, ns). However, noncompleters had histories that could be indicative of a poorer prognosis. Sixty-eight percent of the noncompleters have a substance abuse diagnosis compared to only 42% of the completers (*χ*^2^(1) = 7.06, *p* < .01). Noncompleters had significantly more total arrests prior to entry into the jail diversion program, (6.90 versus 3.15 total arrests; *t*(122) = 3.85, *p* < .001), more disorderly person's convictions (2.46 versus .81 convictions; *t*(122) = 2.63, *p* = .01), and more total jail admissions prior to entering the program compared to completers (4.2 versus 2.2, *t*(122) = 3.8, *p* < .001). In addition, noncompleters had a lower GLOF at program entry (*t*(121) = 2.57, *p* = .013).

Consistent with other research findings, prior history contributes to the explanation of jail diversion program outcomes. In this study, the total number of jail admissions in the follow-up period was predicted by three variables in a multiple regression equation (multiple *r*(3,120) = .52, *p* < .05). These include (1) number of lifetime jail admissions prior to entering jail diversion: more lifetime admissions prior to diversion predict more postdiversion admissions (*β* = .39); (2) whether the individual completed the jail diversion program: completers were incarcerated less after diversion (*β* = −.24); and (3) age: younger people were more likely to be incarcerated more times after diversion (*β* = −.22). As indicated by the beta weights, the best predictor was number of previous jail admissions, followed by program completion.

Using the dichotomous outcome variable of being incarcerated once or more in the follow-up period (1) or not incarcerated at all in the follow-up period (0), there were similar findings to those above. Program completion was the strongest predictor of incarceration followed by the number of days spent incarcerated during the 12 months prior to diversion as the second strongest predictor. Using logistic regression, forward stepwise method, both number of jail days in the prior year and program completion predicted whether someone was jailed or not on any occasion after diversion (*χ*^2^(2) = 41.79, *p* < .001). Each additional day of prior jail admissions increased the likelihood of any postdiversion admission by 2.8% (odds-ratio = 1.028). Having completed the prescribed course of treatment reduced any instance of reincarceration by 82% (odds-ratio = .18). Thus, degree of previous criminal justice involvement is associated with poorer outcomes and, in some cases, explains more of the variance in outcomes than program participation.

## 4. Discussion and Conclusions

Jail diversion services facilitated by a county prosecutor's office and coordinated with community providers result in decreased time in jail and prison, fewer and later arrests, and increased global level of functioning for individuals with serious mental illness. These changes were seen in both completers of the program and noncompleters but were more pronounced among those who had completed their prescribed care. The decreases in days incarcerated and number of arrests are comparable to that of previous studies. The findings suggest that early involvement in jail diversion services is advisable before individuals develop a cycle of numerous arrests and periods of incarceration (McNeil & Binder, 2007). Because completion of the jail diversion program is so strongly associated with more positive outcomes, assertive outreach and other attempts to keep service recipients actively participating are recommended.

The best outcomes were found in the first follow-up year. By the fifth year of follow-up even among “completers” there is an increase in arrests. Thus, there is some question about the sustainability of gains after participation in jail diversion services. While we saw that the number of days incarcerated continues to decline over time, the hazard function for arrests and jail days seems to be rising for the completers later in the follow-up period, particularly in terms of disorderly persons arrests. Among those who completed the program, the risk of arrests peaks in the fourth year of follow-up (36–48 months). In addition, the cumulative proportion of persons still not arrested at 60 months is almost equal to that of noncompleters, although their incarceration days and incidence of incarceration remain significantly lower.

Individuals with a longer length of participation in active treatment had better outcomes. This suggests that a longer length of participation and/or greater continuity of care with other community mental health services are needed. This could take the form of a jail diversion service that is longer in length or a follow-up community service with seamless continuity to the jail diversion program. Both groups (program completers and noncompleters) had their sharpest gains while still actively enrolled in the service and completers who received services for a longer length of time sustained these gains longer. In large part, this is not a surprising finding in light of other treatment services' research for persons with serious mental illness. All of the more effective, evidenced-based community services including assertive community treatment (ACT), supportive housing, and supported employment are long-term. They can provide several years of support, though often support is tapered off as need diminishes. Support can also be increased if need reemerges. Treatment gains seen when enrolled in ACT, in particular, rapidly diminish if the service is prematurely withdrawn.

### 4.1. Strengths and Limitations

This study's findings are based on a nonexperimental design. Its pre-post and longitudinal features are similar to that of other jail diversion studies. Its follow-up period of up to 5 years was longer than most studies, which typically focus on the first 12 months and occasionally continue up to 24 months after diversion. The data analyzed were not collected prospectively but were based upon existing data sets. The post hoc grouping of completers versus noncompleters has its limitations because of the high degree of self-selection and other factors such as preexisting differences that contribute to whether someone remains in the program. In fact, completers and noncompleters had significant differences even before entering the service, with noncompleters having more extensive criminal justice histories and a higher likelihood of having a substance abuse diagnosis. Indeed, some of these so-called criminogenic features, including multiple jail admissions and substance abuse, are predictive of outcomes regardless of jail diversion program participation. This, too, is consistent with previous studies. Thus, this naturalistic design, an evaluation of a program as implemented over several years of operation, provides evidence of the generalizability of these approaches in “real world” and “routine” circumstances.
